# Case Report: Acute common peroneal nerve injury after posterior lumbar decompression surgery

**DOI:** 10.3389/fsurg.2024.1329860

**Published:** 2024-02-12

**Authors:** Peng Wei Wang, Ming Hsuan Chung, Dueng Yuan Hueng, Chung Ching Hsia

**Affiliations:** ^1^ Department of Surgery, Taoyuan Armed Forced General Hospital, Taoyuan, Taiwan; ^2^ Department of Neurological Surgery, Tri-Service General Hospital and National Defense Medical Center, Taipei, Taiwan

**Keywords:** perioperative peripheral nerve injury, common peroneal nerve neuropathy, intraoperative neuromonitoring (IONM), prone position, spine surgery adverse events

## Abstract

Spine surgery is a prevalently performed procedure. Some authors have proposed an age-related surge in surgical and general complications. During spine surgery, patients are placed in positions that are not physiologic, would not be tolerated for prolonged periods by the patient in the awake state, and may lead to complications. Understanding these uncommon complications and their etiology is pivotal to prevention and necessary. The patient is a 76-year-old woman referred to the outpatient department of neurosurgery in February 2022 by her physiatrist with a chief complaint of chronic low back pain and numbness over the left leg. Lumbar spine magnetic resonance imaging revealed degenerative disc disease and posterior disc bulging at the levels of L2/3∼L5/S1 with compression of the thecal sac. After receiving anti-inflammatory medication, nerve block and caudal block, her symptoms persisted. She was referred to a neurosurgeon for surgical intervention. We diagnosed spinal stenosis with left L3 and L4 radiculopathy, and elective decompression surgery was scheduled a few days later. We performed discectomies at L2/3 and L3/4 and left unilateral laminectomy at L2 and L3 for bilateral decompression. Following an uneventful surgery, the patient was extubated, and her left leg pain improved, but pain over the right outer calf with drop foot developed. A second lumbar MRI the next day revealed no evidence of recurrent disc herniation or epidural hematoma. Then, she received nerve conduction velocity and needle electromyogram on postoperative day 2, and the studies indicated right common peroneal nerve entrapment neuropathy. After medication with steroids and foot splint use, right leg pain improved. However, weak dorsiflexion of the right ankle persisted. We referred this patient to a physiatrist and OPD for follow-up after discharge. Perioperative peripheral nerve injury (PPNI) is most commonly caused by peripheral nerve ischemia due to abnormal nerve lengthening or pressure and can be exacerbated by systemic hypotension. Any diseases affecting microvasculature and anatomical differences may contribute to nerve injury or render patients more susceptible to nerve injury. Prevention, early detection and intervention are paramount to reducing PPNI and associated adverse outcomes. The use of intraoperative neuromonitoring theoretically allows the surgical team to detect and intervene in impending PPNI during surgery.

## Introduction

Spine surgeries are among the most commonly performed procedures. Some authors have proposed an age-related surge in surgical and general complications. Proper patient positioning is crucial in spine surgery to ensure optimal operating conditions and exposure of the operative site. During spine surgery, patients are placed in positions that are not physiologic and would not be tolerated for prolonged periods in the awake state, potentially leading to complications (1). While the incidence of complications related to patient positioning in spine surgery is relatively low, peripheral nerve injuries caused by positioning have profound and life-changing effects to patients and their families (2). Understanding the etiology of these uncommon complications and is essential for prevention and necessary intervention.

## Case presentation

**Figure 1:**
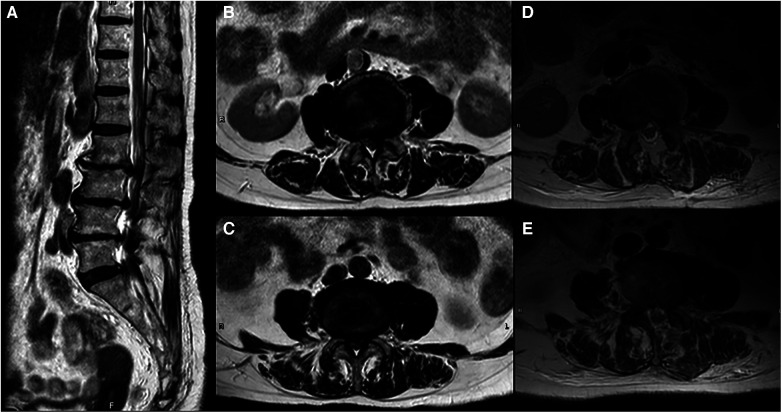
(**A**) Sagittal plane of preoperative MRI shows spondylosis, marginal osteophytes, and degenerative changes of the disk spaces with desiccation and posterior herniation at the levels of L2/3∼L5/S1 which cause compression of the thecal sac. (**B**) Axial plane of preoperative MRI at L2/3 level shows central canal stenosis and left lateral recess stenosis. (**C**) Axial plane of preoperative MRI at L3/4 level shows severe central canal stenosis and bilateral lateral recesses stenosis. (**D**,**E**) Axial plane of postoperative MRI at L2/3, L3/4 level show no evidence of recurrent disc herniation or epidural hematoma.

The patient is a 76-year-old woman (height: 156 cm, weight: 58 kg, Body Mass Index: 23.83) referred to the outpatient department of neurosurgery in February 2022 by her physiatrist with a chief complaint of chronic low back pain and numbness over the left leg. Symptoms commenced in October 2021, leading her to seek assistance at a rehabilitation clinic. Neurological examinations revealed claudication (normal pulsations over bilateral dorsalis pedis artery), dysesthesia over left L3 and L4 dermatomes and straight leg raising test (SLRT) of left lower limb < 60 degrees. Additionally, normal hip flexion, knee extension, ankle dorsiflexion, and plantar flexion of bilateral lower limbs (muscle power: 5), as well as normal patellar, Achilles reflexes were observed. Lumbar spine x-rays indicated degenerative joint disease with osteophyte formation and mild spondylolisthesis over L2/3 and L5/S1. Lumbar spine magnetic resonance imaging (MRI) revealed degenerative disc disease and posterior disc bulging at the levels of L2/3∼L5/S1 with compression of the thecal sac (Figures 1A–C). Despite receiving anti-inflammatory medication, nerve block and caudal block, her symptoms persisted. Consequently, she was referred to a neurosurgeon for surgical intervention. The diagnosis was spinal stenosis with left L3 and L4 radiculopathy, and elective lumbar decompression surgery was scheduled a few days later.

**Figure 2:**
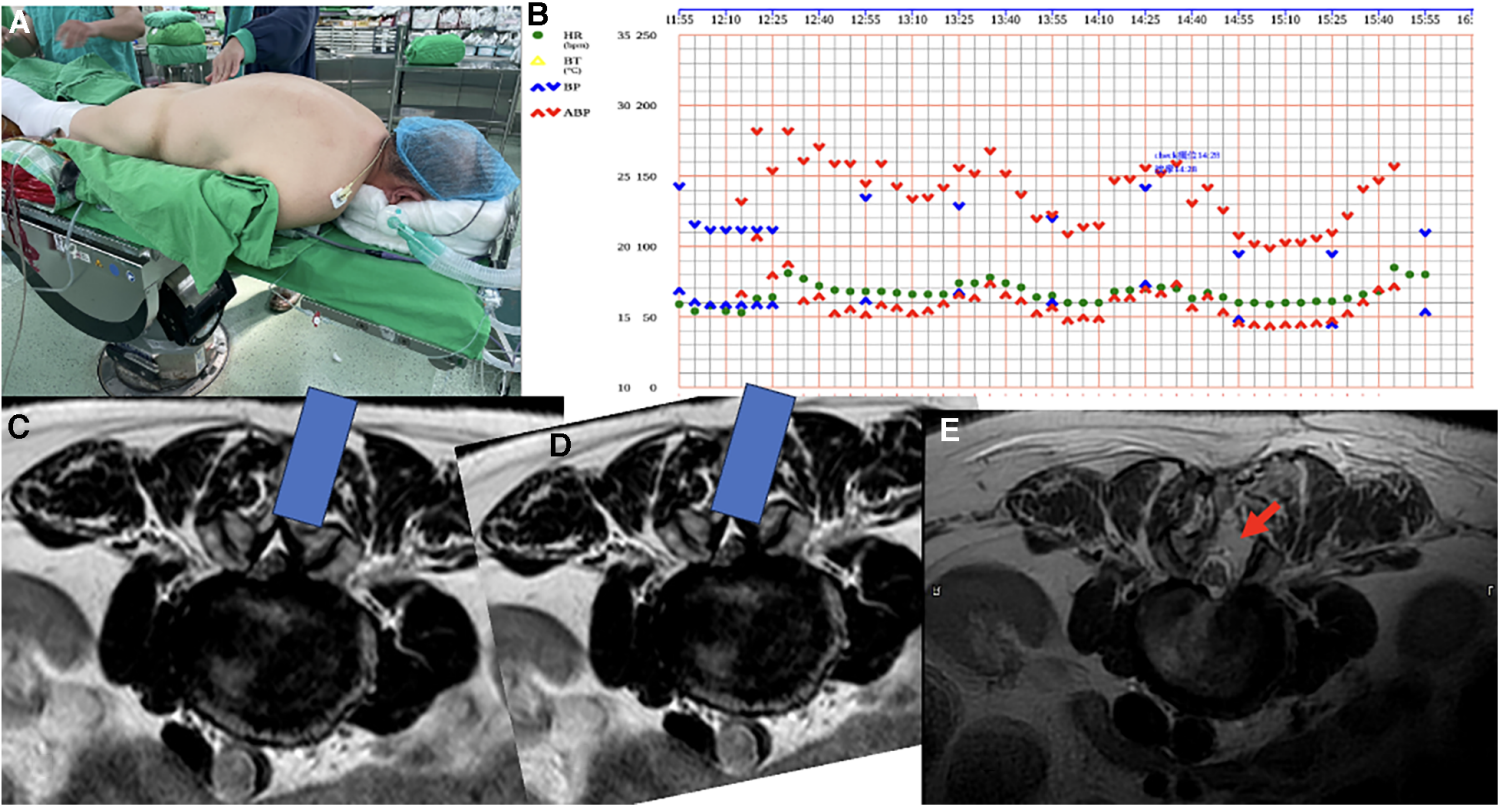
(**A**) The patient was placed in the prone position with upper limbs tucked aside and 6-inch elastic bandages were properly wrapped over bilateral lower limbs from foot to above knee as compression stockings. (**B**) Invasive arterial blood pressure was recorded every 5 min and no systolic blood pressure was less than 100mmHg. Non-invasion arterial blood pressure was checked every 30 min during operation and no systolic blood pressure was less than 90mmHg. (**C**,**D**) Axial plane of pre-operative MRI at L2/3 level, the blue bar indicates the tubular retractor and the view through microscope. Tilting table to the opposite side and undercutting of the spinous process to gain access to the contralateral lateral recess. (**E**) Axial plane of postoperative MRI at the L2/3 level; the red arrow indicates the surgical access, which appears as a high signal on the MRI.

The procedure was performed under general anesthesia, and preoperative evaluation classified this patient as ASA II. The patient was positioned in the prone position with upper limbs tucked aside, and two bolsters were longitudinally placed beneath trunk (Figure 2A). Standard skin was sterilization and surgical draping were carried. A linear incision was made over a 2-cm paramedian region and then wound was deepened to lumbar fascia. Dissection proceeded along the spinal process to expose lamina of L2, L3. A tubular retractor was placed and lower edge of lamina was exposed. Unilateral laminectomies of L2, L3, along with medial partial facetectomy without damaging the fact capsule were performed under a microscope. The Maquet Magnus operating table was tilted about 10–15 degrees to the right side to facilitate bilateral decompression of ligamentum flavum (Figures 2C–E). Smooth discectomies, L2/3 and L3/4 over left side assisted by a root retractor, and left unilateral laminectomies for bilateral decompression (ULBD) were conducted (3). According to anesthesia records, invasive arterial blood pressure was monitored every 5 min and no systolic blood pressure dropped below 100 mmHg (Figure 2B). Following an uneventful surgery lasting 2 h and 42 min, the patient was extubated. Although her left leg pain improved, she developed pain over the right lateral calf with drop foot. A positive Tinel's sign was noted over lateral surface of right fibular neck, along with sensory impairment over right lateral calf and dorsum of foot was also noted. Normal bilateral knee flexion and extension, plantar flexion (muscle power: 5), and weak eversion and dorsiflexion of right ankle (muscle power: 0) were observed. Additionally, an examination of deep tendon reflexes revealed normal patellar and Achilles reflexes.

After receiving medication with steroids and neuropathic pain management, along with the use of a foot splint, the right leg pain improved. However, weak eversion and dorsiflexion of the right ankle persisted (muscle power: 0). We referred this patient to a physiatrist for a rehabilitation program and outpatient department (OPD) follow-up after discharge. This patient could walk with the assistance of an ankle-foot-orthosis but there was no improvement in weak eversion and dorsiflexion of the right ankle (muscle power: 0) after one year of follow-up.

### Diagnostic assessment

**Figure 3:**
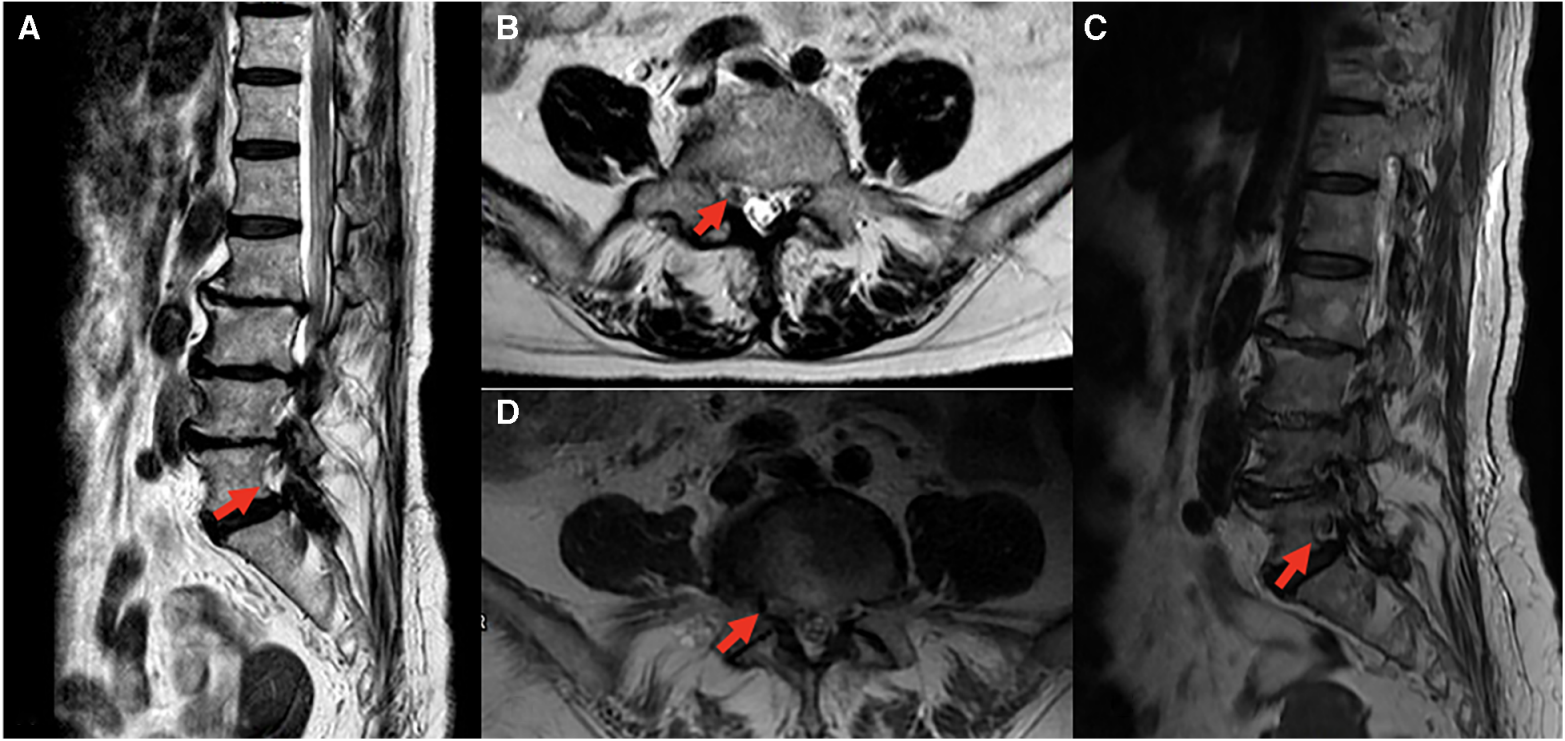
Comparison of preoperative and postoperative MRI, red arrow indicates right L5 root on sagittal and axial planes. All images revealed no significant change or foraminal stenosis of right L5 root. (**A**) Sagittal plane, (**B**) axial plane at L4-5 level of preoperative MRI; (**C**) sagittal plane, (**D**) axial plane at L4-5 level of postoperative MRI.

The postoperative lumbar MRI on the next day revealed no evidence of recurrent disc herniation or epidural hematoma (Figures 1D,E). To differentiate from L5 radiculopathy, often caused by disc bulging at L4/5 level or L5/S1 foraminal stenosis (4), pre- and post-operative lumbar spine MRI were thoroughly examined (Figure 3). Subsequently, the patient underwent nerve conduction velocity (NCV) and needle electromyogram (EMG) on postoperative day 2 (Table 1), revealing reduced amplitudes of compound motor action potentials (CMAPs) in right peroneal nerve and tibial nerve. Some spontaneous activity, such as fibrillation, was observed in the right gastrocnemius muscle and poor motor unit action potentials (MUAPs) occurred in the right tibialis anterior (TA) muscle with complete interference during maximal effort. Reduced amplitudes of CMAPs indicate an axonal loss and spontaneous activity indicates a chronic axonal lesion. The severity of neuropathy and sites of lesion could not be graded or pinpointed based on these electrophysiologic findings.

**Table 1:** NCV post-op day 2: reduced amplitudes of CMAPs on right peroneal nerve & right tibial nerve. The *F*-wave latencies do not exceed *F*-estimates and a relatively normal sensory NCV test of sural nerve. NCV after 6 months: the right peroneal nerve and right sural nerve showed no response. *F* Wave studies indicate that the right peroneal *F* wave has no response. The right tibial motor nerve showed prolonged distal onset latency and reduced amplitude. All H Reflex left vs. right side latency differences were within normal limits. EMG post-op day 2: at rest, there were some spontaneous activities such as fibrillation at right gastrocnemius muscle. In volition, there were poor MUAPs occurring at right TA muscle with complete interference during maximal effort. EMG after 6 months: PSWs and fibrillations were noted at right TA muscle and peroneus longus muscle. Absent MUAPs were noted at right TA muscle.

Nerve conduction studies					Electromyography					
Nerve	Latency difference	Velocity	Amplitude	Segment	Muscle	Nerve	Root	Fibs	PSW	Amplitude
Post-op day 2 Peroneal. R	7.1 ms	42 m/s	2.777 mV	Ankle-fibula head (Ext Dig Bre)	Post-op day 2 Vastus lateralis. R	Femroal	L2-4	None	None	Normal
					Tibialis anterior. R	Deep fibular	L4-5	None	None	0
Tibial. R	8.2 ms	40 m/s	2.448 mV	Ankle-popliteal fossa (Abd Hall Brev)	Gastrocnemius. R	Tibial	S1-2	1+	None	Normal
					After 6 months Tibialis anterior. R	Deep fibular	L4-5	2+	3+	0
Sural. R	2.5 ms	56 m/s	11 uV	Ankle-Calf	Gastrocnemius. R	Tibial	S1-2	None	None	200–5,000 uV
After 6 months Peroneal. R	NR				Peroneal Long. R	Superficial fibular	L5-S1	2+	2+	200–5,000 uV
Sural. R	NR				L4 Paraspinal. R	Rami	L4	None	None	200–5,000 uV
Tibial. R	8.4 ms	43 m/s	1.6 mV	Ankle-popliteal fossa (Abd Hall Brev)	L5 Paraspinal. R	Rami	L5	None	None	200–5,000 uV
					S1 Paraspinal. R	Rami	S1	None	None	200–8,000 uV

Thorough electrophysiologic studies (Table 1) were repeated six months after the onset of palsy, revealing acute denervation signs, such as positive sharp waves and fibrillations noted at right TA muscle and peroneus longus muscle. Absent MUAPs were observed in the right TA muscle, and there was no response in the conduction studies of the right peroneal nerve. The diagnosis of right common peroneal neuropathy (Seddon classification: axonotmesis; Sunderland grade 3–4) is consistent with above findings and clinical presentation, although chronic lumbosacral radiculopathy may be concurrent or a predisposing factor.

## Discussion

Patient positioning is a crucial first step in lumbar surgery, providing access to the cause of stenosis or radiculopathy. Lumbar surgery often requires positions that are not physiologic for an awake patient and maintained for an extended duration. Such positions can lead to advent events due to abnormal forces exerted on the patient's anatomy (1). In a study, the overall incidence of position-related upper extremity somatosensory evoked potential (SSEP) changes during spine surgery was 6.1% (5). The lateral decubitus and prone superman positions were notably associated with more frequent occurrences compared to other operative positions during spine surgery. Prolonged surgery or improper positioning could potentially result in perioperative peripheral nerve injury.

The incidence of perioperative peripheral nerve injury (PPNI) in a retrospective study covering various surgeries during a 10-year period in a tertiary center ranged from 0.03% to 0.1% (2). Neurosurgical and orthopedic procedures showed a significant correlation with perioperative peripheral nerve injury. Factors such as female sex, old age (>65 y/o), hypertension, tobacco use, diabetes mellitus and higher BMI significantly increased the risk (2, 6, 7).

Ischemia is the most likely mechanism in PPNI, as the blood flow to peripheral nerves does not fluctuate with even major changes in blood pressure, rendering peripheral nerves more susceptible to systemic hypotension (1). In addition to the potentially harmful impact of systemic hypotension on peripheral nerves (8), positioning-related factors can exacerbate ischemic damage. Direct pressure on peripheral nerves is the primary mechanism causing neural dysfunction and ischemia. Another latent yet crucial mechanism is peripheral nerve stretching beyond its resting length. An experimental study noted that stretching peripheral nerves by as little as 6%–11% could result in impaired signal transmission due to mechanical injury and circulatory disturbance. Most peripheral nerves cannot tolerate stretching beyond 10% of their normal length (9).

In the context of predisposing factors, several clinical studies suggest that essential hypertension is a potential risk factor for peripheral neuropathy in patients with type I and type II diabetes mellitus (10). Rat models with hypertension exhibited conditions such as nerve ischemia, heightened sensitivity to heat-induced pain, slowed nerve conduction, and the degeneration of nerve fibers. The presence of thinly myelinated fibers accompanied by excess Schwann cells, indicating repeated cycles of demyelination and remyelination, was observed. Additionally, a decrease in the levels of myelin basic protein in the nerves was noted (11).

The common peroneal nerve (CPN), also known as the common fibular nerve, is the smaller and terminal branch composed of the posterior divisions of L4, L5, S1, and S2. Near the distal thigh, just above or in the popliteal fossa, the sciatic nerve divides into the tibial and CPNs. The CPN passes around the neck of the fibula, where it is most superficial and vulnerable to pressure or trauma. After entering the lateral leg compartment deep to the peroneus longus tendon, the CPN divides into deep and superficial peroneal branches (12).

Common peroneal nerve palsy could occur in any surgery adopting the lateral decubitus position because it is adjacent to the bony prominence of the fibular head. Several studies support that prolonged duration in the lithotomy position is associated with a higher risk of lower extremity neuropathy (13). Knee eversion and degenerative varus deformity could also lead to common peroneal nerve injury due to overstretching (14). Some case reports have proposed that postoperative common peroneal nerve palsy is related to prolonged surgery and systemic hypotension during the operation (15).

Due to the lack of clinical studies and the rarity of perioperative lower extremity neuropathy, it is challenging to determine a specific mechanism of nerve injury. We hypothesize that multiple factors, including patient positioning (prone position and tilted to the right side when performing unilateral laminotomy for bilateral decompression), and systemic hypertension as a predisposing factor, collectively contributed to common peroneal nerve palsy in this presented case. Lumbar MRI before surgery revealed a herniated intervertebral disc at the L4/5 level with mild impingement of the right L4 and L5 roots, which may be another predisposing factor. Other potential causes, such as prolonged surgery, hypotension during surgery, type II diabetes mellitus and higher BMI, did not exist in our case.

The development of perioperative common peroneal neuropathy likely results from the convergence of several risk factors. Distinguishing perioperative common peroneal injury, particularly following lumbar surgery, can be more challenging. Postoperative MRI should be utilized to rule out nerve root injury, epidural hematoma and inadequate decompression. While a detailed medical history and neurological examination, including the SLRT test, Tinel's sign, assessment of sensory impairment dermatomes, and muscle strength evaluation, may offer some insights into the etiology, a conclusive diagnosis should depend on MRI and nerve conduction velocity (NCV) electromyography (EMG).

In the context of prevention, intraoperative neuromonitoring is employed for the early detection of PPNI in patients at an elevated risk of comorbidities, including systemic hypertension, type II diabetes mellitus, extreme body weight, and prolonged surgery. Several studies have highlighted the established role of somatosensory evoked potential (SSEP) monitoring in promptly identifying reversible positioning-related signal changes. Correction of positioning, leading to signals improvement, often results in patients waking up without neurologic deficits (5). It is essential to note that factors beyond patient positioning, such as spine instrumentation, hypoperfusion, hypothermia, anesthetic agents, depth, and operating room noise, can influence SSEP signals. The determination of the sensitivity and specificity of SSEPs for PPNI remains an ongoing investigation, and the efficacy of SSEPs in reducing the incidence of PPNI is yet to be clarified (14, 16).

The severity of peripheral nerve injury significantly influences the outcome, and widely accepted classifications, such as those proposed by Seddon (neuropraxia, axonotmesis, and neurotmesis) and Sunderland (grade 1–5 nerve injuries), are employed. Nerve conduction studies (NCS) and needle electromyography (EMG) constitute pivotal tests in diagnosing PPNI (14). These tests confirm the diagnosis, localize the lesion, determine the injury severity, predict outcome, and determine whether direct surgical repair should be performed. To ensure accurate characterization of the nerve lesion, it is recommended to conduct comprehensive NCS and EMG studies in the fourth week following the event (6). For cases involving peripheral nerve palsy, the initial EMG should be performed within the first five days. Notably, patients with neurapraxia typically exhibit normal conduction 4–6 weeks after injury due to remyelination. In contrast, individuals with axonotmesis and neurotmesis experience the loss of both proximal and distal conduction due to Wallerian degeneration. These studies are pivotal for diagnosing nerve injury, precisely locating the site of injury, and assessing the extent of the damage.

Most perioperative peripheral nerve injuries resulting from positioning or hypoperfusion during surgery are typically categorized as neuropraxia or axonotmesis. Individuals affected by this issue may experience lasting motor deficits or chronic pain. Surgical intervention is seldom necessary for PPNI patients. While conservative treatments, such as orthosis use and physiotherapy, are prevalent for patients with peripheral neuropathy, the therapeutic efficacy for PPNI still requires further verification.

PPNI in spine surgery is a rare yet significant perioperative complication, resulting in considerable patient disability, functional impairment, and the potential for legal consequences. Primarily caused by peripheral nerve ischemia due to abnormal nerve lengthening or pressure, PPNI can be exacerbated by systemic hypotension. Diseases affecting microvasculature and anatomical variations may contribute to nerve injury or increase susceptibility to such injuries. Early detection and intervention, facilitated by intraoperative neuromonitoring (IONM), are crucial in mitigating PPNI and its associated adverse outcomes.

### Patient perspective

1. The prone-related common peroneal neuropathy may develop in lumbar spine decompression surgery, especially when the patient is titled to one side.
2. PPNI may cause by direction compression or peripheral nerve ischemia due to the abnormal nerve lengthening or compression or hypotension.
3. Prevention, early detection and intervention are paramount to reducing PPNI and associated adverse outcomes.

## Data Availability

The original contributions presented in the study are included in the article/Supplementary Material, further inquiries can be directed to the corresponding author.
